# *In silico *analysis of natural compounds targeting structural and nonstructural proteins of chikungunya virus

**DOI:** 10.12688/f1000research.12301.2

**Published:** 2017-12-08

**Authors:** Jaspreet Jain, Anchala Kumari, Pallavi Somvanshi, Abhinav Grover, Somnath Pai, Sujatha Sunil

**Affiliations:** 1 Vector Borne Disease group, International Centre for Genetic Engineering and Biotechnology, New Delhi, India; 2 School of Biotechnology, Jawaharlal Nehru University, New Delhi, India; 3 Department of Biotechnology, Teri University, New Delhi, India; 4 Department of Virology and Immunology, Amity University, Uttar Pradesh, India

**Keywords:** In silico analysis, Chikungunya virus, natural compounds, CHIKV E3 protein, CHIKV Capsid protein, Docking, ADME, Ligand-Protein Interaction

## Abstract

**Background:** Chikungunya fever presents as a high-grade fever during its acute febrile phase and can be prolonged for months as chronic arthritis in affected individuals. Currently, there are no effective drugs or vaccines against this virus. The present study was undertaken to evaluate protein-ligand interactions of all chikungunya virus (CHIKV) proteins with natural compounds from a MolBase library in order to identify potential inhibitors of CHIKV.

**Methods:** Virtual screening of the natural compound library against four non-structural and five structural proteins of CHIKV was performed. Homology models of the viral proteins with unknown structures were created and energy minimized by molecular dynamic simulations. Molecular docking was performed to identify the potential inhibitors for CHIKV. The absorption, distribution, metabolism and excretion (ADME) toxicity parameters for the potential inhibitors were predicted for further prioritization of the compounds.

**Results:** Our analysis predicted three compounds, Catechin-5-O-gallate, Rosmarinic acid and Arjungenin, to interact with CHIKV proteins; two (Catechin-5-O-gallate and Rosmarinic acid) with capsid protein, and one (Arjungenin) with the E3.

**Conclusion:** The compounds identified show promise as potential antivirals, but further *in vitro* studies are required to test their efficacy against CHIKV.

## Introduction

Chikungunya virus (CHIKV) is an alphavirus belonging to the Togaviridae family ^1^. These are small, spherical, enveloped viruses that constitute a positive-sense single-stranded RNA genome of approximately 11.8 kb ^2, 3^. The genome encodes for five structural proteins (Capsid (CP), E3, E2, 6K and E1) and four nonstructural polyproteins (nsP1-4). Recently, CHIKV has spread widely and is the cause of a febrile illness of global concern with the potential to affect millions of people worldwide. As of 2016, Chikungunya fever has been identified in nearly 60 countries ( WHO Chikungunya report; accessed 3 August 2017). Some recent outbreaks have been observed in Africa, Asia, Europe, islands in the Indian and Pacific Oceans, and recently on the Caribbean islands in America ^4– 7^. CHIKV infection is characterized by severe debilitating muscle and joint pain, and polyarthralgia, which persists for about 3–12 months and could last up to 1–3 years ^8– 10^. In some instances, severe CHIKV infection may cause neurological disorders and ocular manifestations ^11– 13^. Other symptoms include headache, myalgia, vomiting and rash ^14, 15^. Until now, there is no effective antiviral treatment, or vaccine, is commercially available for the treatment of CHIKV, and patients are treated symptomatically.

Studies on antivirals for chikungunya generally target the replication machinery (nsP2 and nsP3 proteins) ^16– 21^ and surface receptors responsible for the binding of the virus during endocytosis (E1 and E2 proteins) ^19, 21^. Recent studies have shown that CHIKV is able to affect the central nervous system (CNS) like new world alphaviruses, such as Venezuelan equine encephalitis virus and Eastern equine encephalitis virus. Thus, it is important to evaluate CHIKV as a transition between new and old world viruses. Old world viruses use nsP2 to inhibit transcription of host proteins, whereas new world viruses have developed an alternative mechanism of transcription inhibition that is mainly determined by their CP protein ^22^. Hence, CP could be an important target protein for potential antivirals. Up until now, the other structural protein of CHIKV, E3, has not been evaluated as a target for antivirals till now. E3 is the only protein in the CHIKV genome with a secretory signal.

Alphavirus CP is a multifunctional protein known to act as serine protease for self-cleavage and viral genomic RNA binding. It is also known to bind to other CP molecules during nucleocapsid formation, and interact with viral spike proteins during virion formation and budding ^23^. CHIKV CP is 261 amino acids long protein and has a molecular weight of approximately 30kDa, and contains two major domains. N-terminal domain is positively charged and is involved in non-specific RNA binding, while the C-terminal domain regulates globular protease and acts as a binding site for the spike protein ^24^. In addition, nuclear import export signals are present on the CP’s amino acid terminal, forming immobile aggregations with nsP3 and E2 proteins of CHIKV ^25^.

The structural protein E3 is approximately 6KDa, and is found not to be associated with the mature virion ^2^. It serves as the signal sequence for the translocation of E3-E2-6K-E1 polyprotein into the endoplasmic reticulum, working in a clade-specific manner, and its cleavage from E2 is essential for virus maturation ^26^. E3 also mediates pH protection of E1 during virus biogenesis via interactions strongly dependent on Y47 at the E3-E3 interface ^27^.

In the present study, we performed an *in silico* analysis of protein-ligand interactions of all CHIKV proteins using a natural compound library from MolBase to predict potential antiviral compounds for CHIKV infection. Our analysis predicted three compounds that interacted with CHIKV proteins (two with the E3 protein, and one with the CP), making them potential antiviral candidates against CHIKV.

## Methods

### Target identification and homology modeling

Structures of CHIKV proteins E1, E2, E3, nsP2 and nsP3 were downloaded directly from RCSB Protein Data Bank (PDB). For the rest of the CHIKV proteins, CP, 6K, nsP1 and nsP4, whose structures are unavailable, CHIKV sequences present in NCBI, belonging to ECSA (East/Central/South Africa) genotype were downloaded. These sequences were utilized to form a consensus sequence with MEGA 6 ^28^ using clustalW pairwise multiple alignment algorithm with all parameters set at default. Using these consensus sequences, homologous proteins from the PDB were identified using Protein BLAST ^29^ where the algorithm parameters were as follows: Max target sequences=100, Expect threshold=10 using BLOSUM62 scoring parameters, Gap cost=Existence:11 & Extension:1 with conditional compositional score matrix adjustment. The suitable templates for nsP1 and CP with highest query coverage, sequence identity and lowest E-value were selected for homology modeling. For proteins 6K and nsP4, no templates were available, and thus these structures were created using threading and looping method (see next section).

The template and target sequences of all CHIKV proteins were then aligned using CLUSTALW ^30^. MODELLER (version 9.16) was used to generate homology models ^31^. Further, the homology model having the lowest MODELLER objective function (molpdf) or DOPE or SOAP assessment scores, or the one having highest GA341 score was selected as the best model structures and were further utilized for model validation. Nonstructural protein, nsP4, and the small accessory peptide of structural protein 6K did not have any template in PDB; therefore a threading and looping approach was implemented for them using LOMETS (Local Meta Threading Server) ^32^. Both online server and standalone program present as a module of I-TASSER Suite version 5.1, which provides 3D models by combining alignment scores of template to target of 9 different threading programs (FFAS-3D, HHsearch, MUSTER, pGenTHREADER, PPAS, PRC, PROSPECT2, SP3, and SPARKS-X). All parameters were set as default. All structural and nonstructural CHIKV protein sequences were selected as potential drug targets.

### Validation of homology modeled structures

Generated models were validated using MolProbity-(v4.3.1) ^33^. Ramachandran plot analysis was performed for the best protein models by analyzing the phi (Φ) and psi (Ѱ) torsion angles. To check reliability of the modeled structures, the root mean square deviation (RMSD) was calculated by superimposing it on template protein structure using PyMOL (v1.7.0.0) visualization software ^34^. Consistency between templates and the modeled structures were assessed by ProSA-web ^35^ (online server), a statistical analysis tool of all the proteins structures available at RCSB PDB. Here, a statistical average is obtained over the known structures with the help of combined potentials of mean force from the PDB database.

### Molecular dynamic simulations

Stability of the domain regions of CHIKV protein structures was examined by molecular dynamics (MD) simulation using GROMACS (version 5.0) software package ^36^. Optimized Potential for Liquid Simulations All-Atom ^37^ force field was used to energy minimize the structures. Through this energy minimization, the high-energy intramolecular interactions were discarded. In order to avoid the steric clashes, overall geometry and atomic charges were also optimized. The proteins were kept at the center of the rectangular box, which was filled with SPC water model system to create the same environmental behavior of the molecules. All the atoms of the protein and the boundary of the rectangular box were separated by a minimum distance of 10 Å. 0.01M NaCl was used as a solvent exposure.

The system was further energy minimized without any restraints for 50,000-time steps; the steepest descent having step size of 0.01 ps. Then the system was equilibrated to reach a stable temperature by conducting NVT ensemble. Pressure was further equilibrated by NPT ensemble performance. The long-range electrostatic interactions were calculated by using particle mesh Ewald ^38^ method with a cut-off of 0.9 nm for Vander Waals interactions. All the bonds were constrained by LINCS ^39^, where only the water molecule moves to equilibrate with respect to protein structure keeping protein molecule as static. To couple the system Berendsen thermostat (V-rescale) and Parrinello-Rahman barostat were utilized to maintain the constant temperature (300 K) and pressure (1 bar). Further MD analysis was performed to observe structural changes and dynamic behavior of the protein by calculating RMSD, radius of gyration and root mean square fluctuation (RMSF) along with changes in temperature, pressure, density and total energy.

### Virtual screening and molecular docking

Simulated computational models of CHIKV proteins were prepared and their binding sites were predicted using SiteMap (Version 2.3, 2009, Schrödinger, LLC, New York, USA). These were then used to perform molecular docking. The protein preparation wizard was used to prepare CHIKV proteins and a natural remedies library from MolBase database was prepared using the LigPrep module ^40^. Virtual screening of modeled proteins against a natural remedy library from MolBase was done by using GLIDE module in an Extra Precision (XP) mode (Version 5.5. 2009, Schrödinger, LLC). It produces the minimal ranks of inappropriate poses and determines the appropriate binding energy of the three dimensional (3D) structure of the protein along with a ligand ^41, 42^.

### Analysis and output visualization of drug target and protein

After the completion of molecular docking, the docked poses were listed depending upon the respective docking scores. Glide Score (obtained using GLIDE Module of Schrödinger Software Suite 9.0) was used as an empirical scoring function to predict free energy for ligands binding to the receptor. The structure showing minimum binding energy was filtered and subjected for further analysis. The 3D conformation ligand receptor was analyzed using PyMOL ^34^ and Chimera ^43^ v1.10.1 visualization software.

### Absorption, distribution, metabolism and excretion (ADME) screening and toxicity analysis

Pharmacokinetics properties and percent human oral absorption values were further predicted for the potential lead molecules using QikProp module (Version 3.2, 2009, Schrödinger, LLC) ^44^. Both the physically remarkable descriptors and pharmaceutically admissible properties were predicted for neutralized ligands by QikProp. The program predicts 44 different properties, including log P (octanol/water), % human oral absorption in intestine (QP%) and predicted IC _50_ value for blockage of HERG K+ channels (log HERG). The Lipinski’s rule of five ^45^, an important criteria for oral absorption, was evaluated for the acceptability of the compounds. In addition, admetSAR ^46^ v1.0 was used to calculated various attributes of the drugs, including the blood brain barrier (BBB), human intestinal absorption, Caco-2 permeable, aqueous solubility, P-gp substrate and inhibitor, CYP450 substrate and inhibitor, CYP IP, ROCT, HERG inhibition, and toxicity parameters. For Lipinski score calculations, the ligand in SMILE format was uploaded to QikProp. The physicochemical properties and Lipinski Rule of Five were also analyzed by PERL script, “CalculatePhysicochemicalProperties.pl” of MayaChemTools ^47^.

### Ligand-protein interaction studies

The protein-ligand complex interaction at the atomic level was analyzed using Maestro 11.0 (LLC Schrodinger 2016) ^48^ and LigPlot+ ^49^ v1.4.5. The protein and the docked ligand were merged together and uploaded to Maestro Suite vMaestro 11. Further, the “Ligand Interaction Diagram” option was selected to draw the protein-ligand binding interactions in the 2D visualization workspace.

## Results

### *In silico* protein preparation, homology modeling and validation

**Figure 1.**
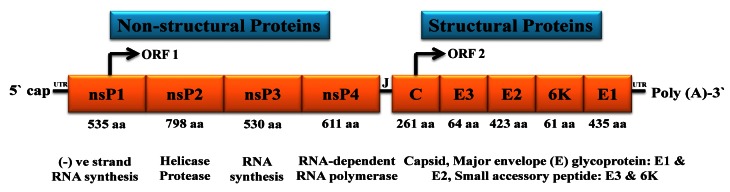
Organization of chikungunya virus genome. The genome consists of two open reading frames (ORFs) separated by an untranslated junction (J). The first ORF encodes for a polyprotein and acts as a precursor of the non-structural proteins (nsP1, nsP2, nsP3 and nsP4). The second ORF encodes the structural proteins (Capsid, E3, E2, 6K and E1). The genome has 5` cap and 3` poly A tail.

**Figure 2.**
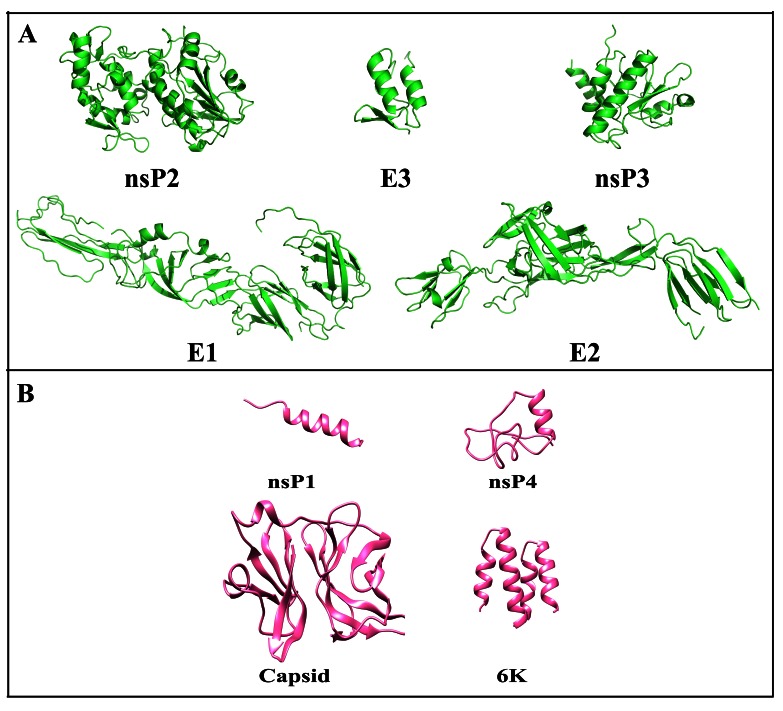
Structures of chikungunya virus proteins. ( **A**) X-Ray structures of nsP2, E3, nsP3, E1 and E2. ( **B**) Homology modelled structures of nsP1, Capsid, nsP4 and 6K.

**Figure 3.**
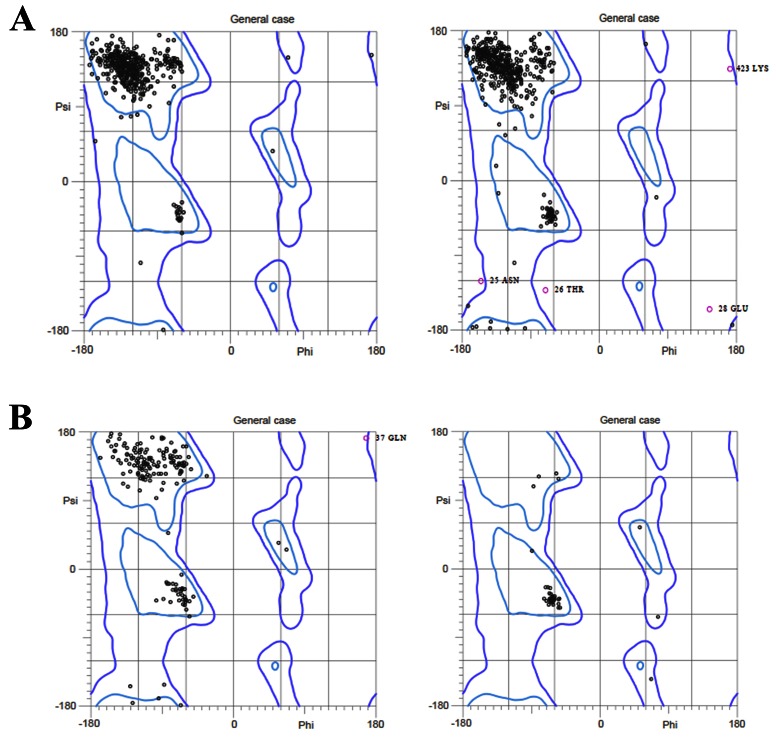
Ramachandran plot of chikungunya virus proteins obtained from MolProbity. ( **A**) nsP1 and Capsid (homology modeling); ( **B**) nsP4 and 6K (Threading/Looping).

CHIKV consists of four nonstructural proteins (nsP1-nsP4), three structural proteins (E1-E3), along with two sub-pro regions 6K and CP, which makes a part of structural protein unit ( Figure 1). Structures of CHIKV proteins E1, E2, E3, nsP2 and nsP3 were downloaded directly from PDB ( Figure 2a), and for other CHIKV proteins (CP, 6K, nsP1 and nspP4) homology modeling and threading and looping methods were utilized to predict their structures. For proteins with templates available, homology modeling was done with five models for every protein created based on sequence similarity using different model generation tools (MODELLER and LOMET), and validated by their internal scoring functions (molpdf, DOPE, SOAP and GA341 scores). Further, ProSA Z-score for all modeled structures were calculated to analyze the quality of models based on the Cα positions. Individual validation and ProSA Z scores for top ranked models are given in Table 1 and their structures are given in Figure 2b. The top ranked models were also analyzed by Ramachandran plot ( Figure 3). The Ramachandran plot shows the distribution of phi (ϕ) and psi (ψ) angles for each amino acid residues of the modeled structures. The respective percentages of the favored and allowed regions for all the residues of all those validated are also shown in Table 1.

**Table 1.** Results for model generation of chikungunya virus (CHIKV) proteins (E3, Capsid, 6K, nsP1 and nsP4). This table includes validation using various simulation scores for the best ranked models for structural and nonstructural proteins of CHIKV.

	TEMPLATE Details		BLAST Results					MODELLAR Results			ProSA Results		Ramachandran plot analysis	
CHIKV Proteins	PDB IDs of the Template	Chain ID	Max Score	Total Score	Query cover	E-Value	Identity	molpdf	DOPE Score	GA341 Score	RMSD (Â)	ProSA Z-Score	Favoured regions (aa residues) (%)	Allowed regions (aa residues) (%)
nsP1	1FW5	A	39.7	39.7	3%	2.00E-04	89%	2441.80	-16203.94	0.70	0.35	0.89	516/533 (96.8%)	532/533 (99.8%)
nsP4	Threading and Looping	-	-	-	-	-	-	-	-	-	0.68	3.04	584/609 (95.9%)	605/609 (99.3%)
Capsid	3J2W	I	315	315	57%	2.00E-110	99%	1253.26	-17861.93	1.00	0.11	-4.17	251/259 (96.9%)	258/259 (99.6%)
6K	Threading and Looping	-	-	-	-	-	-	-	-	-	0.68	-3.05	57/59 (96.6%)	59/59 (100%)

### Molecular dynamic simulation and analysis

**Figure 4.**
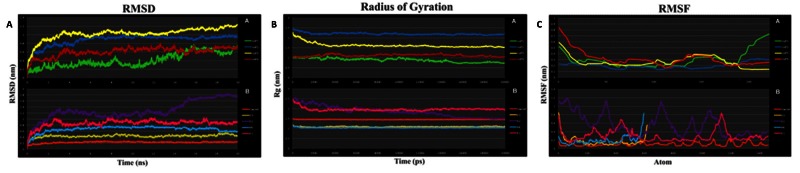
Molecular dynamics profiles of the chikungunya virus (CHIKV) proteins tertiary domain structure optimization. ( **A**) root mean square deviation (RMSD), ( **B**) Radius of Gyration, and ( **C**) root mean square fluctuation (RMSF). **A**– **C** graphs are *vs* Time, and F *vs* Atoms. Each set shows the graph for both Non-structural (upper) and Structural (lower) CHIKV proteins. Non-structural Protein: nsP1 (green), nsP2 (blue), nsP3 (yellow) and nsP4 (red); Structural Proteins: Capsid (orange), E3 (mustard), E2 (purple), 6K (cyan) and E1 (pink).

Molecular dynamic simulations were employed to analyze the protein structure-function complexities, such as structural stability, conformational flexibility and folding. Domain regions of the structures ( Table 2) were simulated for 20 ns. Moreover, various parameters, such as temperature, pressure, density and total energy, were calculated to check the stability of these structures along with steric properties. Further, RMSD values for the backbone atoms of proteins were plotted against time of MD simulations. Average RMSD during the simulations was 22.93. Radius of gyration on the other hand also supports the stability and compactness of the proteins. The RMSF with respect to each residue depicts the flexibility of the proteins. Average RMSF during the simulations was 1.45. The RMSD, radius of gyration and RMSF plots for all CHIKV proteins are shown in Figure 4A–C. The resulting graphs contributed to protein modeling, as they show a constant RMSD deviation throughout the 20ns simulation except for a small deviation in E2 after 14ns. Depending upon these simulation parameters, the proteins showed conformational stability.

**Table 2.** Domain regions/amino acid residues of chikungunya virus (CHIKV) modelled proteins used for molecular docking experiments.

CHIKV	Domain region of protein (residues)
nsP1	245-260
nsP2	28-259
nsP3	28-259
nsP4	2-49
Capsid	113-261
E3	1-64
E2	113-261
6K	1-61
E1	113-261

### Molecular docking

Drug discovery relies heavily on molecular docking to understand the interactions between ligand/inhibitor and target protein ^50^. In this study, we resorted to the docking of available protein structures (wherever applicable), as well validated, refined and simulated modeled proteins to screen against a natural remedy library from MolBase. The binding sites of all protein structures were predicted by SiteMap. The predicted binding pockets were further validated using Glide in XP mode. Top ten ligand/compounds having docking score (Glide Score) above -4, glide energy of -20 kcal mol ^-1^ and potential energy of a considerable range were considered for the next level of screening. The combined results of all the docked ligand along with the glide energy and potential energy have been provided in Table 3. Of these, two ligands, (1,3,6, -Trigalloyl-β-D-Glucose and Quercetin-3-rutinoside (Compound ID 164 and 153) were found to interact with all the proteins and were discarded from further analysis.

**Figure 5.**
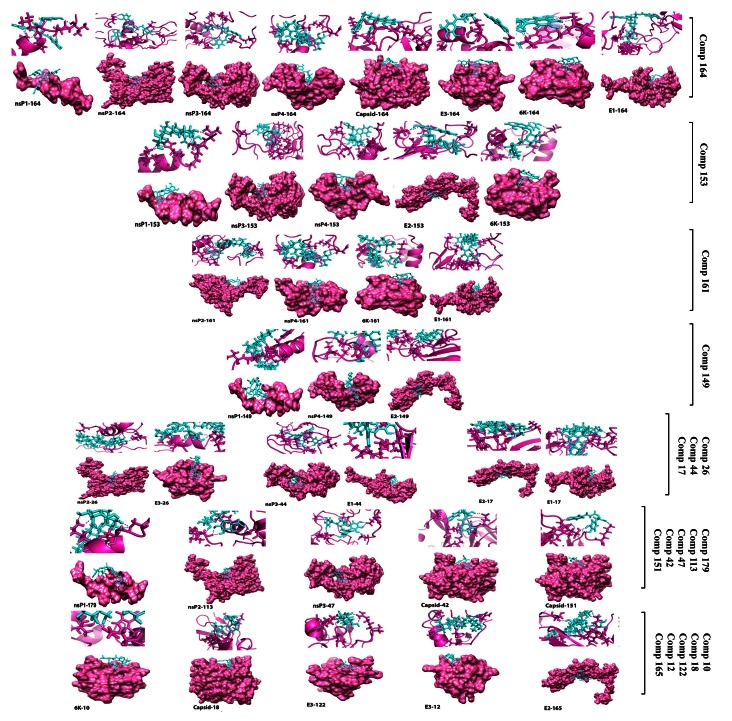
Binding interaction with the potential lead compounds and their representative binding pocket conformation for the top four docked poses of all chikungunya virus proteins. Ligands are cyan sticks and receptors as pink ribbon/surface.

For the non-structural proteins, the top ligands included Rebaudioside A and Withanoside IV (Compound ID 149 and 179) for nsP1; Stevioside, Bacopaside II and Jujubogenin isomer of bacopasaponin C (Compound ID161, 26 and 113) for nsP2; Chebulinic acid and Corilagin (Compound ID 44 and 47) for nsP3; Rebaudioside A and Stevioside (Compound ID 149 and 161) for nsP4. For structural proteins, Catechin-5-O-gallate, Rosmarinic acid and Agnuside (Compound ID 42, 151 and 18) for CP; Bacopaside II, Mangiferin and Arjungenin, (Compound ID 26, 122 and 12) for E3; (Rebaudioside A, Tribulosin and Asiaticoside (Compound ID 149, 165 and 17) for E2; Arjunetin and Stevioside (Compound ID 10 and 161) for 6K; Chebulinic acid, Stevioside and Asiaticoside (Compound ID 44, 161 and 17) for E1. Top four docked poses of the modeled proteins and the small molecules having lowest docking score are shown in Figure 5 (ligand wise).

**Table 3.** Combined results of top four docked ligand with chikungunya virus proteins along with the glide score, glide energy and potential energy.

NON STRUCTURAL PROTEIN						
Comp ID	Compound name	Chemical name	Molecular formula	Glide score	Glide energy	Potential energy
**nsp1**						
164	1,3,6,-Trigalloyl-β -D-Glucose	1,3,6-Trigalloylglucose; β-D-Pyranose-form	C27H24O18	-7.54	-43.61	151.40
149	Rebaudioside A	13-Hydroxy-16-kauren-19-oic acid; entform, 13-O-[β-D- Glucopyranosyl-(1->2)-[β- D-glucopyranosyl-(1->3)]-β -Dglucopyranoside], β-D-g	C44 H70 O23	-6.94	-46.23	622.16
153	Rutin	Quercetin-3-rutinoside	C27 H30 O16	-6.52	-38.91	259.01
179	Withanoside IV	1,3,27-Trihydroxywitha-5,24-dienolide; (1α,3β)-form, 3-O-[β-D-Glucopyranosyl-(1- >6)-β-D-glucopyranoside]	C40 H62 O15	-5.49	-41.46	552.81
**nsP2**						
164	1,3,6,-Trigalloyl-β- D-Glucose	1,3,6-Trigalloylglucose; β-D-Pyranose-form	C27H24O18	-9.47	-64.32	151.40
161	Stevioside	13-Hydroxy-16-kauren-19-oic acid; ent-form, 13-O-[β-D- Glucopyranosyl-(1->2)-α-D-glucopyranoside], β-D- glucopyranosyl ester	C38 H60 O18	-8.66	-45.72	548.61
26	Bacopaside II	Pseudojujubogenin; 3-O-[α-L-Arabinofuranosyl-(1->2)- [β-D-glucopyranosyl-(1->3)]-β-D-glucopyranoside	C47 H76 O18	-7.66	-50.36	955.71
113	Jujubogenin isomer of bacopasaponin C	Jujubogenin; 3-O-[α-L-Arabinofuranosyl-(1->2)-[β-D- glucopyranosyl-(1->3)]-α-L-arabinopyranoside]	C46H74O17	-7.64	-44.88	879.85
**nsP3**						
44	Chebulinic acid	[(3s,3as,4s,7r,8r,10s,11r,17s)-3,15,16-trihydroxy-2,5,13-trioxo- 10,17-bis[(3,4,5-trihydroxybenzoyl)oxy]-8-{[(3,4,5-trihydrox ybenzoyl)oxy]methyl}-2,3,3a,4,5,7,8,10,11,13-decahydro- 7,11-methano[1,4,7]trioxacyclotridecino[11,10,9- de]chromen-4-yl]acetic acid	C41H32O27	-12.36	-82.33	451.70
47	Corilagin	1-O-Galloyl-3,6-(R)- hexahydroxydiphenoyl-β- Dglucopyranose	C27H22O18	-8.96	-56.60	232.70
153	Rutin	Quercetin-3-rutinoside	C27 H30 O16	-8.50	-55.75	259.01
164	1,3,6,-Trigalloyl-β- D-Glucose	1,3,6-Trigalloylglucose; β-D-Pyranose-form	C27H24O18	-8.17	-68.12	151.40
**nsP4**						
149	Rebaudioside A	13-Hydroxy-16-kauren-19-oic acid; entform, 13-O-[β-D- Glucopyranosyl-(1->2)-[β- D-glucopyranosyl-(1->3)]-β- Dglucopyranoside], β-D-g	C44 H70 O23	-8.87	-55.61	622.16
153	Rutin	Quercetin-3-rutinoside	C27 H30 O16	-8.30	-51.12	259.01
164	1,3,6,-Trigalloyl-β- D-Glucose	1,3,6-Trigalloylglucose; β-D-Pyranose-form	C27H24O18	-8.27	-63.20	151.40
161	Stevioside	13-Hydroxy-16-kauren-19-oic acid; ent-form, 13-O-[β-D- Glucopyranosyl-(1->2)-α-D-glucopyranoside], β-D- glucopyranosyl ester	C38 H60 O18	-8.01	-50.24	548.61
**STRUCTURAL PROTEIN**						
**Comp** **ID**	**Compound Name**	**Chemical Name**	**Molecular** **Formula**	**Glide** **Score**	**Glide** **Energy**	**Potential**
**Capsid**						
42	Catechin-5-O- gallate	3,3',4',5,7-Pentahydroxyflavan; (2R,3S)-form, 5-O-(3,4,5- Trihydroxybenzoyl)	C22 H18 O11	-6.26	-38.05	96.39
151	Rosmarinic acid	3-(3,4-Dihydroxyphenyl)-2-hydroxypropanoic acid; (R)-form, 2-O-(3,4-Dihydroxy-E-cinnamoyl)	C18H16O8	-6.12	-28.87	53.75
18	Agnuside	[(1S,4aR,5S,7aS)-5-hydroxy-1-[(2S,3R,4S,5S,6R)- 3,4,5-trihydroxy-6-(hydroxymethyl)oxan-2-yl]oxy- 1,4a,5,7a-tetrahydrocyclopenta[c]pyran-7-yl]methyl 4-hydroxybenzoate	C22H26O11	-5.83	-40.81	188.75
164	1,3,6,-Trigalloyl-β- D-Glucose	1,3,6-Trigalloylglucose; β-D-Pyranose-form	C27H24O18	-5.41	-43.32	151.40
**E3**						
164	1,3,6,-Trigalloyl-β- D-Glucose	1,3,6-Trigalloylglucose; β-D-Pyranose-form	C27H24O18	-6.77	-57.04	151.40
26	Bacopaside II	Pseudojujubogenin; 3-O-[α-L-Arabinofuranosyl-(1->2)- [β-D-glucopyranosyl-(1->3)]-β-D-glucopyranoside	C47 H76 O18	-6.28	-46.22	955.71
122	Mangiferin	2-beta-D-glucopyranosyl-1,3,6,7-tetrahydroxy-9H- xanthen-9-one	C19H18O11	-6.11	-38.93	198.08
12	Arjungenin	2,3,19,23-Tetrahydroxy-12-oleanen-28-oic acid; (2α,3β,19α)-form	C30H48O6	-6.02	-30.81	512.65
**E2**						
149	Rebaudioside A	13-Hydroxy-16-kauren-19-oic acid; entform, 13-O-[β-D- Glucopyranosyl-(1->2)-[β- D-glucopyranosyl-(1->3)]-β- Dglucopyranoside], β-D-g	C44 H70 O23	-10.71	-62.53	622.16
165	Tribulosin	Spirostan-3-ol; (3β,5α,25S)-form, 3-O-[β-DXylopyranosyl- (1->2)-[β-D-xylopyranosyl- (1->3)]-β-D-glucopyranosyl- (1->4)-[α	C55H90O25	-10.07	-60.51	957.86
153	Rutin	Quercetin-3-rutinoside	C27 H30 O16	-8.64	-45.10	259.01
17	Asiaticoside	2,3,23-Trihydroxy-12-ursen-28-oic acid	C48 H78 O19	-8.50	-52.90	805.10
**6K**						
164	1,3,6,-Trigalloyl-β- D-Glucose	1,3,6-Trigalloylglucose; β-D-Pyranose-form	C27H24O18	-6.83	-50.95	151.40
10	Arjunetin	2,3,19-Trihydroxy-12-oleanen-28-oic acid;roxymethyl)oxan- 2-yl] (4aS,6aR,6aS,6bR,10S,11 S,12aS,14bR)-10,11-dihydroxy-12a-(hydroxymethyl)- 2,2,6a,6b,9,9-	C36H58O10	-6.43	-36.56	543.12
161	Stevioside	13-Hydroxy-16-kauren-19-oic acid; ent-form, 13-O-[β-D- Glucopyranosyl-(1->2)-α-D-glucopyranoside], β-D- glucopyranosyl ester	C38 H60 O18	-6.32	-39.69	548.61
153	Rutin	Quercetin-3-rutinoside	C27 H30 O16	-6.06	-40.84	259.01
**E1**						
44	Chebulinic acid	[(3s,3as,4s,7r,8r,10s,11r,17s)-3,15,16-trihydroxy-2,5,13- trioxo-10,17-bis[(3,4,5-trihydroxybenzoyl)oxy]-8-{[(3,4,5- trihydroxybenzoyl)oxy]methyl}-2,3,3a,4,5,7,8,10,11,13- decahydro-7,11-methano[1,4,7]trioxacyclotridecino[11, 10,9-de]chromen-4-yl]acetic acid	C41H32O27	-9.77	-62.87	451.70
164	1,3,6,-Trigalloyl-β- D-Glucose	1,3,6-Trigalloylglucose; β-D-Pyranose-form	C27H24O18	-8.48	-52.97	151.40
161	Stevioside	13-Hydroxy-16-kauren-19-oic acid; ent-form, 13-O-[β- D-Glucopyranosyl-(1->2)-α-D-glucopyranoside], β-D- glucopyranosyl ester	C38 H60 O18	-8.13	-40.94	548.61
17	Asiaticoside	2,3,23-Trihydroxy-12-ursen-28-oic acid	C48 H78 O19	-7.40	-50.53	805.10

Unique ligand-protein partners were taken forward for ADME and toxicity analysis. In case of nonstructural proteins, these ligand-protein pairs were Withanoside IV (Compound ID 179)-nsP1, Jujubogenin isomer of bacopasaponin C (Compound ID 113)-nsP2 and Corilagin (Compound ID 47)-nsP3. In case of structural proteins, these pairs were Catechin-5-O-gallate (Compound ID 42), Rosmarinic acid (Compound ID 151) and Agnuside (Compound ID 18) against CP, Mangiferin (Compound ID 122) and Arjungenin (Compound ID 12) against E3, Tribulosin (Compound ID 165) against E2, and Arjunetin (Compound ID 10) against 6K.

### ADME analysis of all potential leads

ADME screening was performed for all the top hits. Here, 44 various physically remarkable descriptors ^51^ and pharmaceutically admissible properties of the top four lead compounds for every CHIKV protein were calculated using QikPro-P ( Table 4). The Lipinski’s rule of five was further employed to evaluate oral absorption along with ADME. Compounds violating more than 2 Lipinski’s rule of 5 were discarded from further analysis.

**Table 4.** QikProp analysis of physically remarkable descriptors and pharmaceutically admissible properties of unique ligand-protein pairs for chikungunya virus proteins.

	nsP1-179	nsP2-113	nsP3-47	Capsid-42	Capsid-151	Capsid-18	E3-122	E3-12	E2-165	6K-10	Range-95% Drug
**MW**	782.92	899.08	634.46	442.38	360.32	466.44	438.34	504.71	1151.30	650.85	130-725
**SASA**	1066.54	1137.97	803.26	677.81	614.80	741.92	631.99	706.02	1448.98	864.39	300-1000
**FOSA**	697.96	815.05	84.99	46.25	44.58	166.74	94.86	481.89	1000.45	581.88	0-750
**FISA**	337.60	316.73	569.51	353.44	362.96	311.82	374.00	212.11	448.53	277.78	7-330
**PISA**	30.98	6.20	148.76	278.11	207.26	263.36	163.13	12.02	0.00	4.73	0-450
**MV**	2162.23	2381.53	1529.97	1240.78	1082.52	1348.34	1145.40	1448.13	3078.06	1782.28	500-2000
**PSA**	245.37	236.31	322.65	194.44	171.59	185.93	216.29	118.93	337.67	170.47	7-200
**donorHB**	8	9	11	7	5	6	7	5	13	7	0-6
**accptHB**	24.10	26.05	17.85	9.45	7.00	16.35	13.75	8.80	40.60	15.60	(2-20)
**Glob**	0.76	0.76	0.80	0.82	0.83	0.80	0.84	0.88	0.71	0.82	0.75-0.95
**QPpolrz**	69.12	77.66	48.15	40.38	32.08	42.62	34.18	47.42	100.98	58.16	13-70 M
**QPlogPo/w**	-0.20	0.31	-3.21	0.20	0.83	-1.13	-1.87	3.26	-2.92	1.43	(-2-6.5)
**QPlogS**	-4.06	-4.46	-2.93	-3.52	-2.95	-2.75	-2.15	-4.63	-3.23	-4.60	(-6.5-0.5)
**CIQPlogS**	-5.36	-6.49	-5.34	-5.15	-4.23	-2.94	-3.47	-5.66	-5.32	-5.83	(-6.5-/0.5)
**QPlogKhsa**	-1.02	-0.89	-1.10	-0.38	-0.56	-1.12	-1.00	0.29	-2.39	-0.07	(-1.5-1.2)
**QPlogBB**	-4.44	-4.20	-6.16	-3.45	-3.62	-3.44	-3.65	-1.70	-6.94	-2.84	(-3.0-1.2)
**Metab**	13	12	11	9	6	9	8	6	13	9	(1-8)
**QPlogHERG**	-5.59	-5.48	-5.49	-5.71	-3.48	-6.10	-4.94	-1.78	-6.29	-4.43	Below -5
**QPPCaco**	6.23	9.83	0.04	4.41	0.91	10.94	2.81	24.44	0.55	23.00	<25 poor
**QPPMDCK**	2.04	3.35	0.01	1.41	0.32	3.76	0.87	11.39	0.15	8.39	<25 poor
**QPlogKp**	-5.90	-5.61	-10.24	-6.19	-6.42	-5.19	-6.78	-4.71	-7.39	-5.57	(-8/-1)
**RuleOf3**	2	2	2	2	1	2	2	0	2	2	Max 3
**PHOA**	1.12	7.64	0.00	26.71	31.01	13.03	0.00	57.89	0.00	33.76	<25% is poor
**RuleOf5**	3	3	3	1	0	2	2	1	3	2	Max 4

Compounds Catechin-5-O-gallate (Compound ID 42), Rosmarinic acid (Compound ID 151) and Agnuside (Compound ID 18) against CP; Mangiferin (Compound ID 122) and Arjungenin (Compound ID 12) against E3; and Arjunetin (Compound ID 10) against 6K were studied further in greater detail for their toxicity.

### Toxicity analysis

The efficacy and unexpected toxicity of a drug to penetrate biological barriers, such as the intestinal wall or BBB, were considered as a prime determinant of the compounds taken forwards for toxicity tests. CHIKV is an old world virus, but is now seen to affect the CNS as well; therefore, compounds that were predicted to cross the BBB were also considered as potential antivirals. Of all the compounds considered for toxicity analysis using AdmetSAR, Arjunetin (Compound ID 10) was considered ineffective for oral consumption and is also carcinogenic. Also, Agnuside (Compound ID 18) and Mangiferin (Compound ID 122) were not considered as potential antivirals as they are predicted to have positive AMES toxicity ( Table 5).

The compounds that were judged to be potential antivirals were Catechin-5-O-gallate (Compound ID 42) and Rosmarinic acid (Compound ID 151) against CP and Arjungenin (Compound ID 12) against E3 structural protein of CHIKV. Thus, the ligand/drug-protein interaction was studied for these three compounds to understand their interaction pattern and strength of interaction with the protein for their role as potential antivirals against CHIKV ( Table 5).

**Table 5.** AdmetSAR analysis for pharmacokinetics properties, percent human oral absorption values and toxicity determination of drugs/ligands that follow the Lipinski’s rule of five and fulfill other QikProp requirements.

Absorption						
Parameter	18	42	151	10	12	122
BBB	-	-	+	+	+	-
Human intestinal absorption	+	+	+	-	+	+
P-glycoprotein substrate	S	S	S	NS	S	S
P-glycoprotein inhibitor	NI	NI	NI	NI	NI	NI
Renal organic cation transporter	NI	NI	NI	NI	NI	NI
**Metabolism**						
**Parameter**	**18**	**42**	**151**	**10**	**12**	**122**
CYP450 2C9 substrate	NS	NS	NS	NS	NS	NS
CYP450 2D6 substrate	NS	NS	NS	NS	NS	NS
CYP450 3A4 substrate	NS	NS	NS	NS	S	NS
CYP450 1A2inhibitor	NI	NI	NI	NS	NI	NI
CYP450 2C9 inhibitor	NI	NI	NI	NS	NI	NI
CYP450 2D6 inhibitor	NI	NI	NI	NS	NI	NI
CYP450 2C19 inhibitor	NI	NI	NI	NS	NI	NI
CYP450 3A4 inhibitor	NI	NI	NI	NS	NI	NI
CYP Inhibitory Promiscuity	Low	Low	Low	Low	Low	Low
**Toxicity**						
**Parameter**	**18**	**42**	**151**	**10**	**12**	**122**
Human Ether-a-go-go-related gene inhibition	WI	WI	WI	WI	WI	WI
AMES toxicity	AT	NAT	NAT	NAT	NAT	AT
Carcinogens	NC	NC	NC	C	NC	NC
Fish toxicity	HT	HT	HT	LT	HT	HT
Tetrahymena pyriformis toxicity	HT	HT	HT	LT	HT	HT
Honey bee toxicity	HT	HT	HT	HT	HT	HT
Biodegradation	NRB	NRB	NRB	RB	NRB	NRB
Acute oral toxicity	III	III	III	III	III	IV
Carcinogenicity (Three-class)	NR	NR	NR	NR	NR	NR

+: Positive; -: Negative; NS: Non-substrate; S: Substrate; NI: Non-inhibitor; I: Inhibitors; BBB: Blood-brain barrier; CYP450: Cytochrome P450; WI: Weak inhibition; NAT: Non AMES toxic; AT: AMES toxic; NC: Non carcinogens; C: Carcinogen; HT: High toxic; RB: Readily biodegradable; NRB: Not readily biodegradable; NR: Not-required.

### Ligand protein interaction

**Figure 6.**
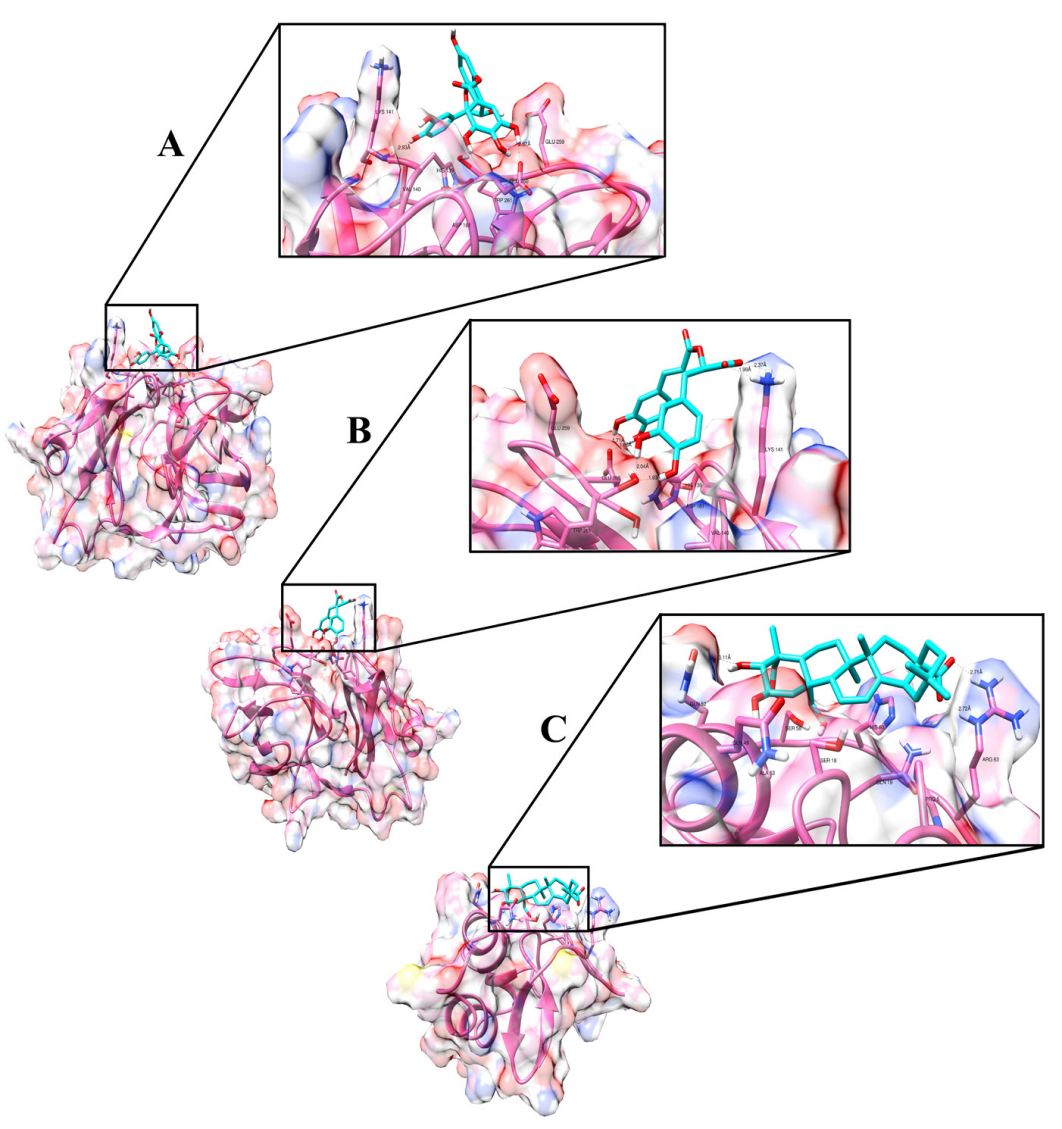
Hydrogen bonding interactions between ligand and chikungunya virus proteins. ( **A**) Hydrogen bonding interaction between Catechin-5-O-gallate [CompID - 42] and capsid, binding affinity of - 6.26 kcal/mol was obtained. The zoomed region shows the receptor-binding pocket. Residues that form hydrogen bond interaction are Glu260 (Distance - 2.57 Å) and Lys 141 (Distance - 2.93 Å); His139, Val140, Asp161, Glu259 and Trp261 forms hydrophobic interaction. ( **B**) Hydrogen bonding interaction between Rosmarinic acid [CompID - 151] and capsid, binding affinity of - 6.11 kcal/mol was obtained. The zoomed region shows the receptor-binding pocket. Residues that form hydrogen bond interaction are Glu260 (Distance - 1.71 and 1.66 Å), Trp261 (Distance - 2.04 and 1.93 Å) and Lys 141 (Distance - 2.37 and 1.99 Å); His139, Val140, Asp161 and Glu259 forms hydrophobic interaction. ( **C**) Hydrogen bonding interaction between Arjungenin [CompID - 12] and E3, binding affinity of - 6.01 kcal/mol was obtained. The zoomed region shows the receptor-binding pocket. Residues that form hydrogen bond interaction are Gln52 (Distance - 3.11 Å) and Arg63 (Distance - 2.72 and 2.71 Å); Pro5, Ser18, Gln19, Gln49, Ala53, Ser58 and His60 forms hydrophobic interaction.

A ligand protein interaction study was done for validated protein structures as discussed earlier. CP residues (Peptidase S3 domain) were predicted to bind to Catechin-5-O-gallate and Rosmarinic acid (Compound IDs 42 and 151, respectively) and E3 residues (Endopeptidase domain) bind to Arjungenin (Compound ID 12). The top docking conformation of Catechin-5-O-gallate showed a predicted binding energy of -6.26 kcal mol-1, whereas Rosmarinic acid and Arjungenin showed similar binding energy of -6.11 kcal mol-1 and -6.01 kcal mol-1, respectively. The binding energy (Glide Score) and the interaction energy (Potential, Vander Waals and Electrostatic) are shown in Table 3. The intermolecular hydrogen bonds and hydrophobic residues showing close contact between receptor proteins (CP and E3) and ligand (Compound ID 42, 151 and 12) are shown in Table 6 and Figure 6A–C, respectively.

**Table 6.** Intermolecular hydrogen bonds and hydrophobic residues showing close contact between receptor chikungunya virus proteins and ligand.

Compound	Interacting Residue	H Bond Distance (Å)	H Bond (D-H--A)	Hydrophobic Residues
**Catechin-5-O-gallate**	Capsid:Glu260:OE1 - UNK900:het O4	2.567	HOE1-H--O4	His139, Val140, Asp161, Glu259, Trp261
	Capsid:Lys141:N - UNK900:het O9	2.927	HN-H--O9	
**Rosmarinic acid**	Capsid:Trp261:O1 - UNK900.het H14	2.039	HO1-H--H14	His139, Val140, ASP161, Glu259
	Capsid:Trp261:O1 - UNK900.het H15	1.927	HO1-H--H15	
	Capsid:Lys141:2HZ - UNK900:het O8	2.375	2HNZ-H--O8	
	Capsid:Lys141:3HZ - UNK900:het O5	1.987	3HNZ-H--O5	
	Capsid:Glu260:OE1 - UNK900:het H4	1.712	HOE1-H--H4	
	Capsid:Glu260:OE1 - UNK900:het H5	1.658	HOE1-H--H5	
**Arjungenin**	E3:Arg63:HNE - UNK900:het O6	2.720	HNE-H--O6	Pro5, Ser18, Glu19, Gln49, Ala53, Ser58, His60
	E3:Arg63:HN2 - UNK900:het O2	2.707	HN2-H--O2	
	E3:Gln52:OE1 - UNK900:het O1	3.108	HOE1-H--O1	

**Figure 7.**
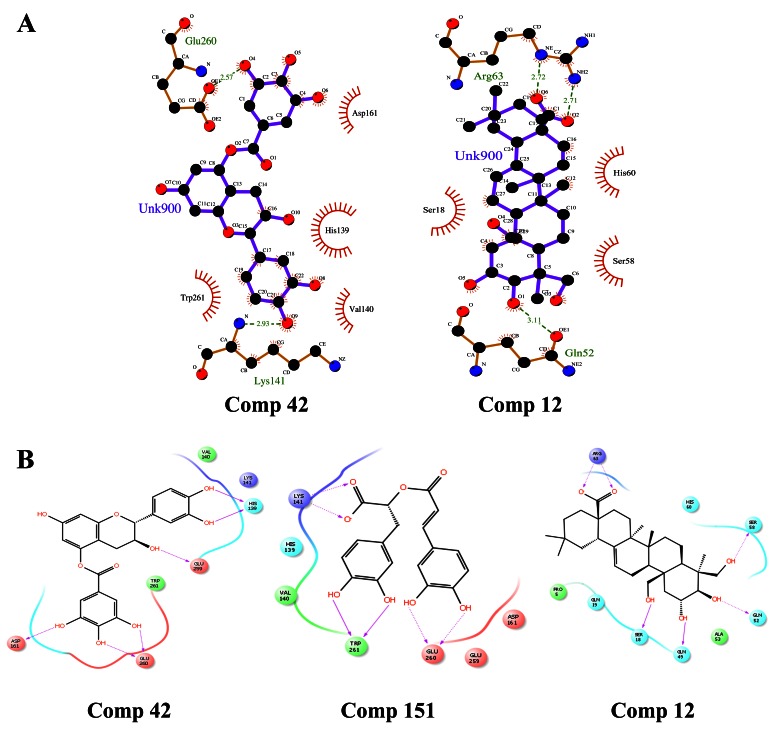
Intermolecular hydrogen bonding in 2D view. ( **A**) LigPlot of Comp 42 (Capsid) and Comp 12 (E3). ( **B**) Maestro ligand interaction diagram of Comp 42 and 151 (Capsid) and Comp 12 (E3).

The interaction result showed that most of the hydrogen bond donors are from the protein that bind to the acceptors on the respective ligands. The compound Catechin-5-O-gallate (Compound ID 42) binds to Glu260 and Lys141 residues (HBond distance of 2.57 and 2.93 Å) of the CP protein and forms hydrophobic interactions with Asp161, His139, Val140 and Trp261 residues ( Figure 7a). Further 2-D workspace revealed that when the ligand-protein interactions were observed both in the presence and absence of solvent the compound Catechin-5-O-gallate binds to the CP protein, HIS139 forms the hydrogen backbone; GLU259, GLU260, ASP161 form the hydrogen side chain. The ligand forms hydrophobic interactions with TRP261, VAL140, LYS141 ( Figure 7b). We were unable to acquire the Ligplot for the interaction of Rosmarinic acid (Compound ID 151) with CP protein as the coordinates were undetectable; however, using 2-D workspace, we identified that Rosmarinic acid binds to the CP protein, TRP261 forms the hydrogen backbone; GLU260, LYS141 form the hydrogen side chain. The ligand forms hydrophobic interactions with HIS139, VAL140, GLU259, ASP161 ( Figure 7b). The third compound Arjungenin (Compound ID 12), binds with Arg63 and Gln52 residues (HBond distance of 2.72 and 3.11 Å) of the E3 protein and Ser18, His60 and Ser58 residues are involved in hydrophobic interactions ( Figure 7a). Its 2-D workspace revealed that SER18, GLN49 form the hydrogen backbone; GLN52, SER58, ARG63 form the hydrogen side chain. The ligand forms hydrophobic interactions with PRO5, GLN19, ALA53, HIS60 ( Figure 7b). Overall docking and interaction results for the best three natural compounds have been compiled in Table 7.

**Table 7.** Overall docking and interaction results for best three natural compounds.

Comp ID	Compound Name	Interacting CHIKV protein	Docking Score	Binding Energy (Kcal/mol)	Number of H-bond interaction	Residues in molecular interaction	Hydrophobic Residues
42	Catechin-5- O-gallate	Capsid	-6.26	-38.05	2	Glu260, Lys141	Asp161, His139, Val140, Trp261
151	Rosmarinic acid	Capsid	-6.11	-28.87	6	Lys141, Glu260, Trp261	His139, Val140, ASP161, Glu259
12	Arjungenin	E3	-6.01	-30.81	2	Gln52, Arg63	Ser18, His60, Ser58

## Discussion

Several drug candidates have been tested for their antiviral activity against CHIKV ^8, 52, 53^. Recent studies have employed chemical libraries to screen for drug candidates for chikungunya with limited success ^16, 17^. Recent efforts for identifying natural compounds against alphavirus replication revealed 44 inhibitors that were effective against several alphaviruses, including CHIKV replicon and Sindbis virus. The study revealed that these compounds inhibited the early stages of viral replication ^19^. Currently, hundreds of thousands of natural compounds are available that can be utilized for screening purposes for identifying novel drug targets. The present study was performed using virtual screening of a natural compound library from MolBase, which showed three compounds, namely, Catechin-5-O-gallate, Rosmarinic acid and Arjungenin, as promising potential antivirals against CHIKV proteins.

Previous studies have suggested that Catechin-5-O-gallate is the most important catechin in green tea, commonly known as epigallocatechin-3-gallate (EGCG). Other catechins are also found in green tea extract, such as epigallocatechin, epicatechingallate and epicatechin. The biological activity of EGCG is assumed to be contributed by the galloyl side chain ^54^. EGCG is known to have antiviral activities towards a variety of viruses. EGCG also inhibits the cell entry of several viruses, such as human immunodeficiency virus (HIV) ^55– 57^ influenza virus ^58^ and hepatitis C virus (HCV) ^59– 61^. Additionally, inhibitory effects of EGCG on viral transcription have been described for viruses like hepatitis B virus, adenoviruses, or herpes viruses ^62^. In case of CHIKV, a recent study on EGCG showed inhibition of CHIKV transduction by blocking cell entry against env-pseudotyped lentiviral vectors, which inhibits CHIKV attachment ^63^.

Rosmarinic acid (RA), a phenolic compound found in various Labiatae herbs ^64^, possesses several properties, such as anti-inflammatory ^65, 66^ and antioxidative, as it reduces liver injury induced by *d-galactosamine* ^67^ and lipopolysaccharides ^68^. Besides these, RA acts as a potent antiviral agent against Japanese encephalitis virus (JEV), another alphavirus closely related to CHIKV. The study indicated that RA reduced viral replication within the brain along with the secondary inflammation resulting from microglial activation. These observations suggested that RA exhibited efficient antiviral as well as anti-inflammatory activity against Japanese equine encephalitis virus infection and hence was able to reduce disease severity ^66^.

The compound Arjungenin, a popular triterpenoid isolated from *Terminalia arjuna*/ *T. chebula*, shows inhibitory effects on HIV-1 Protease ^69, 70^. Arjungenin has been previously used for a wide range of activities that includes anti-inflammatory, anti-microbial, anti-cancer and even anti-viral ^71^, but no work has been done on this particular natural compound to date.

## Conclusion

Treatment of chikungunya includes antipyretic drugs during the febrile stage and depends heavily on symptomatic relief during the chronic arthritic phase. Our present study has identified natural compounds that may be antiviral and might be good candidates as drugs for chikungunya treatment. Further *in vitro* validation is required for these compounds to provide insights into their mode of action against the different stages of chikungunya infection.

## Data availability

All source data relating to this article can be found in Supplementary File 1.

## Supplementary material

**Supplementary File 1:** Structures of CHIKV proteins (E1, E2, E3, nsP2 and nsP3) downloaded from PDB; sequences of the unknown structures found in NCBI (CP, 6K, nsP1 and nsP4); the homologous proteins identified for the unknown structure proteins in PDB and modeled structures using these template structures; protein structures prepared by threading and looping methods; the natural remedy library from MolBase and Ligand-protein docked structures.
