# Supplementary material for: In silico analysis of natural compounds targeting structural and nonstructural proteins of chikungunya virus
Source: F1000Res. 2017 Dec 8;6:1601. Originally published 2017 Aug 30. [Version 2] doi: 10.12688/f1000research.12301.2 (PMC5747330; doi:10.12688/f1000research.12301.2)
Supplement: Supplementary file 1 [file f1000research-6-14481-s0000.tgz › c487a9a0-f221-4db5-bc33-7996efe2df6c.zip!/Supplementary File 1/Acession numbers and Molbase url.docx]

| **Genbank Accessions** | **Genbank Accessions** | **Genbank Accessions** | **Genbank Accessions** | **Genbank Accessions** |
| --- | --- | --- | --- | --- |
| AB455493 | FJ000068 | FJ513673 | GU301780 | JN558835 |
| AB860301 | FJ000069 | FJ513675 | GU301781 | JN558836 |
| AY726732 | FJ445426 | FJ513679 | HE806461 | JQ067624 |
| DQ443544 | FJ445427 | FJ807897 | HM045791 | JX088705 |
| EF012359 | FJ445428 | FJ807898 | HM045792 | KJ451624 |
| EF027134 | FJ445430 | FJ807899 | HM045793 | KJ679577 |
| EF027135 | FJ445431 | FN295484 | HM045797 | KJ679578 |
| EF027136 | FJ445432 | FN295485 | HM045800 | KJ796844 |
| EF027137 | FJ445433 | FN295487 | HM045804 | KJ796845 |
| EF027138 | FJ445443 | FR717336 | HM045805 | KJ796846 |
| EF027141 | FJ445445 | FR717337 | HM045808 | KJ796847 |
| EF452493 | FJ445463 | GQ428210 | HM045810 | KJ796848 |
| EU037962 | FJ445484 | GQ428211 | HM045811 | KJ796849 |
| EU244823 | FJ445502 | GQ428212 | HM045820 | KJ796850 |
| EU372006 | FJ445510 | GQ428213 | HM045821 | KJ796851 |
| EU564334 | FJ445511 | GQ428214 | HM045822 | KJ796852 |
| EU703762 | FJ513628 | GQ428215 | HM045823 | FJ513657 |
| FJ000062 | FJ513629 | GQ905863 | HQ456251 | GU301779 |
| FJ000063 | FJ513632 | GU189061 | HQ456252 | JN558834 |
| FJ000064 | FJ513635 | GU199350 | HQ456253 |  |
| FJ000065 | FJ513637 | GU199351 | HQ456254 |  |
| FJ000066 | FJ513645 | GU199352 | HQ456255 |  |
| FJ000067 | FJ513654 | GU199353 | JF274082 |  |

GenBank Accession Ids of all CHIKV nucleotide sequences (ECSA clade) used to make consensus sequences

MolBase Url: <http://www.molbase.com/en/cas-registry-number.html>

**Note: From the above mentioned url, only the natural compounds were considered for this study and the ligands were prepared using Schrodinger’s Ligand preparation pipeline.**
