# Supplementary material for: In silico analysis of natural compounds targeting structural and nonstructural proteins of chikungunya virus
Source: F1000Res. 2017 Dec 8;6:1601. Originally published 2017 Aug 30. [Version 2] doi: 10.12688/f1000research.12301.2 (PMC5747330; doi:10.12688/f1000research.12301.2)
Supplement: Peer review history

# Peer review: Referee response for version 2

**DOI:** 10.5256/f1000research.14481.r28833

**Published:** 2017-12-18

**Authors:** Soma Chattopadhyay

**Affiliations:** 1 Infectious Disease Biology, Institute of Life Sciences, (Autonomous Institute of Department of Biotechnology, Government of India), Bhubaneswar, Odisha, India

There are two points I want to mention. These are as follows:

**Use of control in molecular docking:** Docking score was used to filter the compounds as antiviral. Since docking score does not exactly estimate the binding free energy, decoys (control) should have been employed for comparisons to support the selection.

**Calculation of ligand efficiency (LE):** There is lack of clarity regarding author’s explanation about LE. LE is usually defined as the average free energy of binding in kcal/mol per non-hydrogen atom ^1^. It is fine as it is in the manuscript.

I have read this submission. I believe that I have an appropriate level of expertise to confirm that it is of an acceptable scientific standard.

## References

[1] MurrayCWErlansonDAHopkinsALKeserüGMLeesonPDReesDCReynoldsCHRichmondNJ: Validity of ligand efficiency metrics. *ACS Med Chem Lett*.2014;5(6) : 10.1021/ml500146d 616-8 10.1021/ml500146d 24944729PMC4060940

# Peer review: Referee response for version 1

**DOI:** 10.5256/f1000research.13316.r27576

**Published:** 2017-11-02

**Authors:** Debasis Nayak, Parimal Kar

**Affiliations:** 1 Centre of Biosciences and Biomedical Engineering, Indian Institute of Technology Indore, Indore, Madhya Pradesh, India; 2 Indian Institute of Technology Indore, Indore, India

**Overall comment:** The article in the present form needs some revision including through checking of grammar and sentence structure. Further, I would request the authors to consider following points. 

1. Authors have written that "All bonds were constrained by LINCS". This claim needs to revisit. Generally, bonds involving hydrogen atoms are restrained using *LINCS*. 
2. Authors have mentioned that "In order to avoid the steric clashes, overall geometry and atomic charges were also optimized". I do not think authors have optimized the atomic charges. Rather atomic charges were obtained from the OPLS forcefield that was used in the simulations.
3. The cutoff used for PME was 0.9 Angstrom. This is very small. It should rather be 9 Angstrom.
4. Berendsen thermostat with V-rescale is not a good option for conformational sampling. A better option is Langevin dynamics. 
5. Authors have written that "The system was further energy minimized without any restraints for 50,000-time steps; the steepest descent having step size 0f 0.01 ps". This claim needs to revisit. No time-step is required for energy minimization. Only to study dynamics, i.e, to integrate Newton's equation of motion, we need to set time-step.
6. It was not mentioned what time-step was used for the heating stage and production simulation.
7. Docking is not a good method for predicting the binding free energy. It is the least accurate method for estimating the binding free energy. However, it is a very good method for predicting the binding pose. Authors should have conducted MM-PBSA type analysis to estimate the binding free energy.
8. Figure 4 A, B, C are not required.
9. Looking at Figure 4D, one can see that the for a couple of cases, longer simulation is required. The simulation length is too small (20 ns) to investigate the dynamics of proteins. They should extend all simulations to 50 ns.
10. Figure 4: Use bigger fonts. It's not readable.
11. Calculate average RMSD and Radius of Gyration during the simulations.
12. Authors have written that "RMSF with respect to each residue....". RMSF is calculated with respect to the average structure obtained from simulations. Do you use C-alpha atoms of each residue?

We have read this submission. We believe that we have an appropriate level of expertise to confirm that it is of an acceptable scientific standard.

# Response

**Published:** 2017-12-04

**Authors:** Sujatha Sunil

**Affiliations:** International Centre for Genetic Engineering and Biotechnology, New Delhi, India

**We thank the reviewers for their valuable comments and suggestions. We have considered all their suggestions and have incorporated them in the updated version of the manuscript. Point by point replies to their queries are mentioned below.** 

- Authors have written that "All bonds were constrained by LINCS". This claim needs to revisit. Generally, bonds involving hydrogen atoms are restrained using *LINCS*. 

**Answer:** Depending on the time-step applied, setting a constraint to bonds is required. The time-step depends on the highest frequency of motion within the system. If a time-step superior to 1 fs is chosen (as in our study, we have chosen 2 fs time-step), bonds involving hydrogen will not be sampled sufficiently, thus they should be constrained. Here, we have constrained all-bonds even heavy atom-H bonds by LINCS algorithm.

- Authors have mentioned that "In order to avoid the steric clashes, overall geometry and atomic charges were also optimized". I do not think authors have optimized the atomic charges. Rather atomic charges were obtained from the OPLS forcefield that was used in the simulations.

**Answer: **The initial structure of the protein in optimized and the atomic charges of the protein are calculated by using the OPLS force field.

- The cutoff used for PME was 0.9 Angstrom. This is very small. It should rather be 9 Angstrom.

**Answer: ** We apologize for the confusion. The cutoff used for PME was 0.9 nm, Angstrom has been replaced by nm in the revised MS. 

- Berendsen thermostat with V-rescale is not a good option for conformational sampling. A better option is Langevin dynamics. 

**Answer: **Berendsen thermostat was used to re-scale the velocities of particles in molecular dynamics simulations to control the simulation temperature. No conformational sampling was done in the study. 

- Authors have written that "The system was further energy minimized without any restraints for 50,000-time steps; the steepest descent having step size 0f 0.01 ps". This claim needs to revisit. No time-step is required for energy minimization. Only to study dynamics, i.e, to integrate Newton's equation of motion, we need to set time-step.

**Answer: **Time step (nsteps) of 50000 i.e the maximum number of (minimization) steps to perform was provided during energy minimization. Below are the parameters provided in mdp file.

 ; minim.mdp - used as input into grompp to generate em.tpr

 ; Parameters describing what to do, when to stop and what to save

 integrator         = steep             ; Algorithm (steep = steepest descent minimization)

 emtol               = 1000.0          ; Stop minimization when the maximum force < 1000.0 kJ/mol/nm

 emstep          = 0.01                 ; Energy step size

 nsteps              = 50000 Maximum number of (minimization) steps to perform

 ; Parameters describing how to find the neighbors of each atom and how to calculate the interactions

 nstlist               = 1                   ; Frequency to update the neighbor list and long range forces

 ns_type                        = grid              ; Method to determine neighbor list (simple, grid)

 rlist                  = 1.0                ; Cut-off for making neighbor list (short range forces)

 coulombtype   = PME             ; Treatment of long range electrostatic interactions

 rcoulomb         = 1.0                ; Short-range electrostatic cut-off

 rvdw                = 1.0                ; Short-range Van der Waals cut-off

 pbc                  = xyz               ; Periodic Boundary Conditions (yes/no)

  

- It was not mentioned what time-step was used for the heating stage and production simulation.

**Answer: **Time-step of 20 ns was used for production simulations. Below is the parameter details provided in mdp file for production simulations.

                                     nsteps = 10000000; 2 * 10000000 = 20000 ps , 20 ns

  

- Docking is not a good method for predicting the binding free energy. It is the least accurate method for estimating the binding free energy. However, it is a very good method for predicting the binding pose. Authors should have conducted MM-PBSA type analysis to estimate the binding free energy.

**Answer: **The study was to screen the natural compounds against CHIKV structural and non-structural proteins and identify potential inhibitors. MM-PBSA for a large number of compounds was out of the scope of the present study and hence not pursued. 

- Figure 4 A, B, C are not required.

**Answer:** We abide by the reviewer’s recommendation. The figures have been removed and changes figure is attached to the revised manuscript.

- Looking at Figure 4D, one can see that the for a couple of cases, longer simulation is required. The simulation length is too small (20 ns) to investigate the dynamics of proteins. They should extend all simulations to 50 ns.

**Answer:  **Except for E2, every simulation has reached to a stable state as shown in the RMSD graph.

- Figure 4: Use bigger fonts. It's not readable.

**Answer: **We apologize for this inconvenience. The figure has been changed.

- Calculate average RMSD and Radius of Gyration during the simulations.

**Answer: **RMSD Average - 22.93

 Radius of Gyration Average - 1.45 

- Authors have written that "RMSF with respect to each residue....". RMSF is calculated with respect to the average structure obtained from simulations. Do you use C-alpha atoms of each residue?

**Answer: **As one can see in graph 4F, the x-axis represents the atomic positions in the trajectory after fitting to a reference frame.  Yes, C-alpha atoms of each residue have been used for the analysis.

# Peer review: Referee response for version 1

**DOI:** 10.5256/f1000research.13316.r25511

**Published:** 2017-09-12

**Authors:** Soma Chattopadhyay

**Affiliations:** 1 Infectious Disease Biology, Institute of Life Sciences, (Autonomous Institute of Department of Biotechnology, Government of India), Bhubaneswar, Odisha, India

The present study by Jain *et al *entitled “ ***In silico *analysis of natural compounds targeting structural and nonstructural proteins of chikungunya virus**” was undertaken to evaluate protein-ligand interactions of all chikungunya virus (CHIKV) proteins with natural compounds from a MolBase library in order to identify potential inhibitors of CHIKV. The authors have predicted three compounds, Catechin-5-O-gallate, Rosmarinic acid and Arjungenin, to interact with CHIKV proteins; two(Catechin-5-O-gallate and Rosmarinic acid) with capsid protein, and one (Arjungenin) with the E3 which required further *in vitro *studies to test their efficacy against CHIKV.

 The study is designed and executed properly, however there are few points which need to be addressed before consideration.

 The points are as follows: 

1. While considering drug like properties, authors have discarded molecules with 2 violations. Why did they not discard compounds with one violation?
2. No control was used in molecular docking?
3. Since the ligands were filtered mainly based on their docking score, ligand efficiency should have been taken as the criteria for selection.
4. From the final three compounds, at least catechin-5-O-gallate and rosmarinic acid are associated with poor absolute oral bioavailability. Then how they can be advanced as drug candidates?

I have read this submission. I believe that I have an appropriate level of expertise to confirm that it is of an acceptable scientific standard, however I have significant reservations, as outlined above.

# Response

**Published:** 2017-12-04

**Authors:** Sujatha Sunil

**Affiliations:** International Centre for Genetic Engineering and Biotechnology, New Delhi, India

**We thank the reviewer for her valuable comments and suggestions. Point by point replies to their queries are mentioned below.** 

  

- While considering drug like properties, authors have discarded molecules with 2 violations. Why did they not discard compounds with one violation?

**Answer:** One violation (20% violation) was kept as it showed better results for toxicity and thus was allowed.

- No control was used in molecular docking?

**Answer:** Docking is an *insilico* process and defined by parameters and not controls. Thus, no control was taken for the study.

- Since the ligands were filtered mainly based on their docking score, ligand efficiency should have been taken as the criteria for selection.

**Answer: **Ligand efficiency is calculated by the binding energy as well as h-bonds of ligand and protein and is also included in the results **. **

- From the final three compounds, at least catechin-5-O-gallate and rosmarinic acid are associated with poor absolute oral bioavailability. Then how they can be advanced as drug candidates?

**Answer: **All these compounds were positive for human intestinal absorption for oral bioavailability based on our in silico toxicity assays and thus could be advanced as drug candidates if we pursue the studies. However, to avoid confusion based on Caco permeability data that showed negative for these compounds, we have removed this data from the revised manuscript.
